# From Insight to Impact: Spotlight on the Potential Implications of the Neuronal Nitric Oxide Synthase Isoenzyme (nNOS) in Experimental Chronic Toxoplasmosis

**DOI:** 10.3390/diseases14070240

**Published:** 2026-07-03

**Authors:** Marwa Omar

**Affiliations:** Department of Medical Parasitology, Faculty of Medicine, Zagazig University, Zagazig 44519, Egypt; moabdelaziz@medicine.zu.edu.eg; Tel.: +20-1114324769

**Keywords:** *Toxoplasma gondii*, brain tissue, nNOS, 7-Nitroindazole, L-arginine, chronic toxoplasmosis, nitric oxide

## Abstract

Background/Objectives: The coccidian protozoan *Toxoplasma gondii* (*T. gondii*) is among the most prevalent zoonotic parasites worldwide. Nitric oxide (NO) production by macrophages is considered a critical microbicidal mechanism against various intracellular pathogens, including *T. gondii*. While the role of the inducible nitric oxide synthase isoenzyme (iNOS) has been widely investigated in both acute and chronic *T. gondii* infections, the specific functions of the neuronal (nNOS) isotype in the antiparasitic immune response, particularly during chronic toxoplasmosis, remain largely uncovered. Hence, this report seeks to bridge the gap regarding the potential participation of nNOS in experimental chronic *T. gondii* infection. Methods: The study included 56 Swiss albino mice, equally allocated into four experimental groups: (G1) negative control, (G2) infected control, (G3) infected-L-arginine-treated, and (G4) infected-7-Nitroindazole-treated. All groups except (G1) were orally infected with the avirulent (ME49) *T. gondii* strain. Nine weeks post-infection, all mice were euthanized for parasitological, histopathological, immunohistochemical, and biochemical analyses. Results: The NO donor, L-arginine, induced a significant reduction in the number of *T. gondii* cysts, together with strong nNOS immunoreactivity in the brain sections of the treated mice. Conversely, the highest parasitic burden was observed following selective nNOS inhibition with 7-Nitroindazole, exacerbating parasite-induced pathology. Conclusions: The neuronal isotype serves as a critical source of NO production during the chronic stage of *T. gondii* infection, thereby enhancing parasite elimination and contributing to host tissue protection.

## 1. Introduction

*Toxoplasma gondii* (*T. gondii*) is a coccidian parasite belonging to the phylum Apicomplexa. This obligatory intracellular protozoan is regarded as one of the most successful pathogens, as it is capable of infecting and propagating in all nucleated cell types, with a special predilection for reticuloendothelial cells [1]. Toxoplasmosis, caused by *T. gondii,* is a widespread zoonotic disease of significant morbidity and mortality, affecting approximately one-third of the world’s human population [2]. Despite its impact, toxoplasmosis remains a neglected parasitic infection and represents the third leading cause of the health burden from foodborne illnesses [3].

*Toxoplasma gondii* exhibits a broad host range, with felids serving as the only definitive hosts and all warm-blooded animals, including humans, acting as intermediate hosts. Human infection typically occurs through ingestion of tissue cysts in undercooked meat or consumption of food and water contaminated with oocysts. Additional transmission routes include congenital transfer, organ transplantation, and blood transfusion.

The life cycle of *T. gondii* is sustained through three biological stages of infection, namely tachyzoites, sporozoites, and bradyzoites. Tachyzoites are rapidly multiplying forms responsible for the acute phase of toxoplasmosis. After a few multiplication cycles, *T. gondii* tachyzoites convert into bradyzoites, which encyst in several organs, predominantly in the brain and muscle tissues [4]. Chronic toxoplasmosis remains subclinical in healthy individuals. However, tissue cyst reactivation can be clinically disastrous, causing Toxoplasma encephalitis (TE), a severe and potentially fatal condition in immunocompromised patients who are prone to lethal activation of dormant cysts [5,6].

Among the several effector immune mechanisms described against *T. gondii* infection, the role of nitric oxide (NO) has also been reported. Activation of the high-output NO pathway in macrophages is preferentially driven by a Type I immune response, characterized by substantial IFN-γ production [7,8].

Nitric oxide (NO) is a diatomic free radical whose duality in action enables both beneficial signalling and potential oxidative damage. This key effector molecule plays complex regulatory roles in physiology and pathophysiology [9], in addition to its anti-parasitic effects against both metazoa and protozoa [10]. Nitric oxide (NO) production is regulated by the family of nitric oxide synthases (NOS), which comprises three distinct isoforms: neuronal nNOS (NOS I), inducible iNOS (NOS II), and endothelial eNOS (NOS III). The NOS enzymes catalyze the oxidation of L-arginine to produce NO and L-citrulline [11].

Neuronal NOS and endothelial NOS, both calcium-dependent isoforms, are constitutively expressed at low levels under physiological conditions. In contrast, the calcium-independent iNOS is rapidly induced by Th1 cytokines, such as IFN-γ and TNF-α, which trigger macrophage activation and enhance NO production [8,11].

Neuronal NOS serves as the primary source of NO in both central and peripheral neurons. Within the central nervous system (CNS), NO produced by nNOS contributes to the regulation of blood pressure, in addition to its modulation of several physiological processes, including memory, learning, and neurogenesis [12]. Till now, the anti-parasitic functions of the neuronal enzyme (nNOS) have received less attention. Recently, nNOS proved protective during the enteral phase of experimental trichinosis. Intestinal expression of this isoenzyme enhanced the elimination of adult *Trichinella spiralis* (*T. spiralis*) worms [13]. Conversely, nNOS failed to clear *Heterophyes heterophyes* (*H. heterophyes*) worms from the intestinal sections of infected dogs [14].

In toxoplasmosis, nitric oxide plays a paradoxical role. Despite its reported protective anti-parasitic role during acute *T. gondii* infection, NO induces detrimental and potentially lethal impacts on host tissues, leading to necrosis and degenerative changes in the liver and bowel of infected mice [15]. In chronic toxoplasmosis, the generation of NO maintains control of the established infection. It acts as a critical modulator of long-term immunity against the parasite [16].

Of the three nitric oxide synthase isoforms, the inducible isotype (iNOS) has been extensively studied during both the acute and chronic phases of toxoplasmosis [15,16,17,18]. In contrast, knowledge is only beginning to emerge about the impacts of the neuronal enzyme (nNOS) on *T. gondii* infection. Building on this gap, this research pivoted toward understanding the less-explored activities of nNOS in chronic cerebral toxoplasmosis.

## 2. Materials and Methods

### 2.1. Toxoplasma gondii Strain

The Medical Parasitology Department, Faculty of Medicine, Alexandria University in Egypt, kindly provided the avirulent cystogenic (ME49) *T. gondii* strain. In order to establish chronic toxoplasmosis, the strain was consistently maintained by repeated inoculation of Swiss albino mice every 8 weeks with 0.1 mL of brain homogenate from previously infected mice containing approximately 100 tissue cysts/mL [19]. For experimental infection, mice were orally inoculated with 10 *T. gondii* cysts/0.1 mL/mouse [20].

### 2.2. Animals

Using the Epi-Info software programme version 7, the sample size was calculated to include 56 apparently healthy, laboratory-bred Swiss albino mice at a 95% confidence level and 80% power of test. The animals were 6 to 8 weeks old and weighed between 20 and 25 g each. Only male mice were selected to eliminate variables related to pregnancy and parturition. The mice were sourced from the animal facilities of Theodor Bilharz Research Institute (TBRI), Giza, Egypt. All breeding and experimental procedures were carried out at the Medical Parasitology Department, Faculty of Medicine, Zagazig University, in accordance with institutional and national rules for the care and use of laboratory animals. The mice were kept in individual, well-ventilated cages with the proper constant temperature (25 ± 2 °C), relative humidity (55–65%), and lighting (12 h light/12 h dark cycle). They were fed a commercial meal high in protein and fiber, with free access to water [19]. To exclude parasitic infections, faecal examination was performed [21].

### 2.3. Drugs: Tested Compounds

The current research study was conducted to evaluate the potential impacts of the neuronal isoenzyme nNOS against *T. gondii* infection, using the following treatment lines; the nitric oxide donor, L-arginine that was applied to assess the main source of NO production, and the selective neuronal nitric oxide synthase NOS inhibitor 7-Nitroindazole (7-NI), which was chosen to investigate the potential protective/pathological impacts of nNOS isotypes during chronic toxoplasmosis.

The nitric oxide donor, L-arginine monohydrochloride (A5131, Sigma-Aldrich, St. Louis, MO, USA), was supplied as a powder and dissolved in normal saline before use. Mice received the drug at a dose corresponding to 2% of the total caloric value/animal, representing 10 mg of L-arginine/day, according to Viana et al. [22]. The drug was administered by oral gavage for seven consecutive days prior to infection [13,23].

The selective neuronal nitric oxide synthase (nNOS) inhibitor 7-nitroindazole (7-NI) (Cat. No. N7778) was obtained from Sigma-Aldrich Co. (St. Louis, MO, USA). Due to its poor water solubility, 7-NI was dissolved in dimethyl sulfoxide (DMSO) and administered intraperitoneally to allow efficient delivery to target sites [24]. The drug was given at a dose of 25 mg/kg [25], starting 42 days post-infection and continued for two weeks [18].

### 2.4. Experimental Design

The current experimental study involved 56 mice, which were equally divided into four groups (14 mice each), as follows: (G1) (normal control): non-infected, non-treated mice, each receiving 100 μL of phosphate-buffered saline (PBS) daily via gavage syringe. (G2) (infected control): The infected, untreated group comprised mice that were orally infected with 10 ME49 tissue cysts in 0.1 mL brain suspension per mouse, administered using gavage syringes fitted with blunt nozzles [20]. The third group (G3) included infected mice treated orally with L-arginine supplementation for seven days prior to infection, as described by Zheng et al. [23]. (G4) consisted of infected mice treated with 7-NI following the protocol implemented for the NOS inhibitor in chronic toxoplasmosis [18]. The drug was administered 7-NI for two weeks, beginning 42 days post-infection.

Nine weeks post-infection, the experiment was terminated as planned. Animals from different study groups were anaesthetized with isoflurane, followed by thoracotomy. Blood samples were collected from the chest cavity. For a subsequent biochemical test, serum was isolated and kept at −20 °C. After that, cervical dislocation was used to sacrifice every animal. Brain samples were swiftly extracted and cut longitudinally at the midline. For later histological and immunohistochemical examinations, half of each brain was preserved in 10% formalin. The second half was used for parasitological evaluation [26].

### 2.5. Assessment Measures

#### 2.5.1. Parasitological Evaluation: Enumeration of Tissue Cysts

To prepare a brain suspension, the remaining unfixed brain half from each mouse was homogenized in 1.5 mL of saline (0.9% NaCl) using a mortar and pestle [18,20]. For cyst enumeration, 2 drops (20 μL, each) of brain homogenate were then dispensed onto glass slides, air-dried, and fixed with absolute methanol (Spectrum Chemical MFG. Corp., Compton, CA, USA). After fixation, the films were stained with Giemsa stain. Slides were examined microscopically under X40 objectives to determine cyst numbers. The count obtained was then multiplied by 25 to calculate the total number of tissue cysts per 1 mL (1000 μL) of brain homogenate [19]. Finally, the mean number of tissue cysts was determined for each study group.

#### 2.5.2. Histopathological Assessment

For histology, brain samples from several research groups were fixed in 10% neutral buffered formalin, dehydrated in ascending grades of ethyl alcohol, cleared in xylene, and embedded in paraffin (Embedding Station: Leica EG1160, Labexchange - Laboratory Equipment Exchange GmbH, Burladingen-Hausen, Germany) for further processing. Subsequently, a microtome was used to obtain serial brain sections of 5 μm thickness. The tissue microsections were then de-waxed, rehydrated, and stained with haematoxylin and eosin (H&E) [27]. Stained sections were examined under a light microscope (Olympus, Hamburg, Germany) to compare pathological changes between the tested groups. The inflammatory process was evaluated depending on the protocol implemented by Saad et al. [28]. The intensity of inflammation was scored in a semi-quantitative approach on a scale ranging from (+1 to +4), where +1 indicates mild changes, with less than one inflammatory cell focus per ×100 field, +2 = moderate inflammatory changes, with 1–5 foci/×100 field, +3 = large inflammatory infiltrate, with >5 foci/100× field, and +4 corresponded to extensive inflammation, characterized by a widespread inflammatory reaction throughout the tissue. Five representative fields from each H&E section were examined at ×100, ×200 and ×400 magnifications for histopathological scoring.

#### 2.5.3. Immunohistochemical Assessment

In the current study settings, the avidin–biotin–peroxidase complex (ABC) method was applied to examine the distribution of the neuronal marker (nNOS) in the brain sections of infected mice, as described by Czarnewski et al. [29]. Briefly, 4- to 5-μm paraffin sections were prepared on poly-L-lysine-coated slides, deparaffinized for 5 min in each of three xylene series, and rehydrated through descending grades of ethyl alcohol and distilled water. Sections were deposited in citrate buffer (pH 6.0; Thermo Fisher Scientific Inc., San Diego, CA 92121, USA, Cat. No. 005000) in a microwave oven for 20 min after being treated with tris-buffered saline (TBS) for five minutes in order to retrieve antigens. Sections were treated with 3% hydrogen peroxide in absolute methanol for seven minutes at room temperature in order to inhibit endogenous peroxidase activity. Tissue sections were incubated with the primary antibody, mouse monoclonal anti-nNOS (1:200 dilutions; Santa Cruz Biotechnology Inc., Santa Cruz, CA, USA, Cat. No. sc-5302) according to the manufacturer’s recommendations. Sections were incubated with horseradish peroxidase (HRP)-conjugated mouse anti-rabbit secondary antibody (San-ta Cruz Biotechnology Inc., Santa Cruz, CA, USA, Cat. No. sc-2357) following washing with phosphate-buffered saline (PBS). Diaminobenzidine (DAB) substrate (DAKO Corp., Fremont, CA, USA) was applied for five minutes in order to visualize the reaction. For two minutes, Mayer’s hematoxylin was applied as a counterstain. The same method was used to process negative controls, except that the primary antibody step was omitted [30].

Immunostaining of the nNOS isoenzyme was evaluated semi-quantitatively following the grading scheme of Hao et al. [31], which considers both the intensity and extent of staining. Positive cells were identified by the presence of brownish cytoplasmic colouration. A scale of 0 to 3 was used to measure the strength of the staining; 0 denoted no staining, 1 mild staining, 2 moderate staining, and 3 noticeable intensities. The extent of staining was graded as 0 for less than 5%, 1 for 5–25%, 2 for 25–50%, 3 for 50–75%, and 4 for more than 75% of cells. The intensity and extent values were multiplied to determine the final score, which ranged from 0 to 12. Scores were categorized as high (9–12), moderate (5–8), and negative or low (0–4). The immunoreactivity for nNOS was assessed microscopically in five representative fields for each tissue section using the Leica Qwin 500 C Image Analyzer system (Leica Imaging Systems Ltd., Cambridge, UK).

To minimize observer bias, all histopathological and immunohistochemical tissue sections were randomized, coded, and examined blindly.

#### 2.5.4. Biochemical Assessment: Serum Nitric Oxide (NO) Levels

Concentrations of nitrites (NO_2_^−^), indicative of NO production, were measured in the serum samples of the tested mice using the colourimetric nitric oxide assay kit (Abcam Inc., Waltham, MA, USA, Cat. No. ab65328) following the directions of the manufacturer. A microtiter plate was filled with 85 μL of each serum sample, a set of prepared standards, and a sample blank. There were two steps in the test methodology. In order to convert nitrate to nitrite, 5 μL of each nitrate reductase mixture and enzyme cofactor were added to the sample and standard wells. This was followed by an incubation period of 60 min. The plate was then incubated for ten minutes after 5 μL of the enhancer was applied. To create a deep purple azo molecule from nitrite, 50 μL of each Griess reagent (R1 and R2) was added in the second stage. The amount of the azochromophore reflects the concentrations of NO in the samples. Absorbance was measured at 450 nm. All measurements for the standards, controls, and samples were performed in triplicate and reported in micromolar (μM) units.

### 2.6. Statistical Analysis

IBM SPSS software version 25 (IBM, Armonk, NY, USA) was used to conduct statistical analyses. Standard deviation (SD), mean, and range were used to report numerical data. The analysis of variance (ANOVA) F-test, followed by Tukey’s post hoc analysis, was applied to compare variables across the study groups. The non-parametric Chi-square (χ^2^) test was employed to compare categorical variables [32]. Treatment efficacy was determined using the following formula: Efficacy (%) = 100 × (mean cyst number in controls minus mean cyst number in treated mice) divided by mean cyst number in controls [33]. Statistical significance was defined as a *p*-value less than 0.05 in all analyses [34].

### 2.7. Ethical Statement

The Scientific Research Ethical Committee of the Faculty of Medicine, Zagazig University (Egypt) (ZU–IACUC) approved the study under ethical code (ZU–IACUC/3/F/43/2024). All animal experiments adhered to the research protocols and complied with the recommendations and guidelines of the Declaration of Helsinki. Animals had unrestricted access to food and water. Upon completion of the studies, euthanasia was performed using isoflurane, and all possible measures were taken to minimize their suffering.

## 3. Results

### 3.1. Parasitological Results

#### Parasitic Load

Based on the data presented in Table 1, the highest parasitic burden was observed in group (G4) after 7-NI administration, with a 46.2% increase in mean cyst count compared to the infected, untreated group (G2) (P2: <0.001 **). The study has also evaluated the efficacy of the nitric oxide donor, L-arginine, which induced a significant reduction in the number of *T. gondii* tissue cysts, compared to groups (G2) (P1: <0.001 **) and (G4) (P3: <0.001 **). Examples of the recovered *T. gondii* cysts are depicted in Figure 1.

### 3.2. Histopathological Results

#### 3.2.1. Histopathological Features

The examined brain sections of the control negative group (G1) exhibited normal structural configuration, without haemorrhage, necrosis or inflammatory infiltration changes, as shown in Figure 2 panels a and b). After *T. gondii* infection, the study recorded well-defined tissue cysts in the brain sections of the infected, untreated mice (G2). The infection was also associated with parasite-induced pathological sequelae, including neuronal loss, encephalitis, and extensive lymphocytic infiltrates (Figure 2c,d). Treatment of the infected mice with the nitric oxide donor, L-arginine, in (G3) caused a resolution of pathological features and a restoration of normal tissue architecture (Figure 2e). The drug also induced degeneration of existing tissue cysts (Figure 2f). In contrast, the pathological findings persisted after 7-NI administration in (G4). The treated mice exhibited ventricular dilatation and encephalomalacia (Figure 2g,h). The reported features could be attributed to the notable increase in parasitic load following the selective inhibition of the neuronal isoform (nNOS) using 7-NI.

#### 3.2.2. Inflammatory Changes

This study also assessed the extent of inflammatory changes in the brain sections of the infected groups. As shown in Table 2, more than 40% (42.9%) of *T. gondii*-infected mice exhibited severe inflammation. Furthermore, the degree of inflammatory response worsened following selective inhibition of the neuronal isoenzyme (nNOS) with 7-NI, as none (0%) of the treated mice in (G4) displayed mild or moderate infiltration changes. Conversely, the intensity of inflammation significantly reduced (*p* < 0.001 **) after L-arginine treatment; in (G3), more than 60% (64.3%) of mice showed mild inflammatory changes.

### 3.3. Immunohistochemical (IHC) Results

#### 3.3.1. Neuronal Nitric Oxide Synthase (nNOS) Expression

The present study has evaluated the distribution of nNOS in brain sections from different experimental groups, with brownish cytoplasmic staining indicating positive immunoreactivity. The examined tissues showed different staining intensities of nNOS. Specifically, sections from non-infected, non-treated mice (G1) showed low to moderate nNOS immunostaining (Figure 3a). In contrast, *T. gondii* infection induced a strong nNOS expression surrounding neuronal and microglial brain cells (Figure 3c and Figure 3d, respectively). While pronounced nNOS distribution was observed after L-arginine treatment in (G3) (Figure 3e), the study recorded negative expression for the neuronal marker following its selective inhibition with 7-NI in (G4) (Figure 3f).

#### 3.3.2. nNOS Immunohistochemical Score

According to Table 3, 78.6% of the control negative mice (G1) exhibited negative or low immunostaining for nNOS, whereas 21.4% showed a moderate nNOS reaction in their brain sections. Notably, *T. gondii* infection induced a significant (P1 < 0.05 *) increase in nNOS expression in the infected mice, indicating upregulation of the marker during chronic toxoplasmosis. L-arginine also generated strong nNOS reactivity, with 50% of (G3) mice exhibiting extensive nNOS expressions. After 7-NI administration, a significant reduction (P4 < 0.001 **) in nNOS levels was observed compared to infected untreated mice (G2).

### 3.4. Biochemical Results

At nine weeks post-infection, serum NO levels were measured across different study groups. As shown in Figure 4, *T. gondii* infection induced a significant reduction in NO expression compared to the negative control group (G1) (*p* < 0.001 **). In contrast, the highest serum production of the proinflammatory agent (104.50 Umol/L) was observed in group G3 following administration of the NO donor L-arginine. Selective nNOS blockage in (G4) resulted in the lowest serum NO levels (23.03 Umol/L), showing a significant reduction (*p* < 0.001 **) compared to group (G1).

## 4. Discussion

While the role of the inducible enzyme, iNOS, has been widely investigated in experimental toxoplasmosis [35,36,37,38], limited data are available regarding the participation of the neuronal isotype (nNOS) in the host’s battle against *T. gondii* infection. Therefore, this study is an effort to extend our knowledge about the functional roles of nNOS-derived nitric oxide in chronic toxoplasmosis.

To the best of the author’s knowledge, this study is the first to investigate the potential implications of nNOS inhibition on chronic cerebral toxoplasmosis. Based on parasitological analysis, the nNOS-specific inhibitor, 7-NI, resulted in a significant elevation in the parasitic load, with a 46.2% increase in the mean cyst count (Table 1). These findings suggest that the neuronal enzyme nNOS is essential for controlling chronic *T. gondii* infection.

Current literature on nNOS-derived nitric oxide (NO) in the antiparasitic immune response remains scarce. The documented effects, however, are not uniformly protective. In experimental giardiasis, Li et al. [39] demonstrated that the neuronal isoform nNOS, rather than the inducible form iNOS, is responsible for eliminating *Giardia lamblia* infection; nNOS-deficient mice exhibited delayed parasite clearance, whereas iNOS deficiency did not significantly affect *Giardia* elimination. Similarly, intestinal nNOS expression was essential for the expulsion of *T. spiralis* adult worms [13]. By contrast, in another model, selective nNOS inhibition failed to eliminate *Heterophyes heterophyes* worms from infected puppies [14].

Several studies have demonstrated nNOS expression in response to both viral and bacterial infections. In Border Disease Virus (BDV) infection, strong nNOS expression in neurons and endothelial cells was associated with multiple pathological outcomes, including neuronal degeneration, gliosis, and necrosis in BDV-infected small ruminants [40]. Further investigation into human influenza virus infection reported increased nNOS expression in maternally infected neonatal hippocampal brains [41]. In murine polymicrobial peritonitis, deficiency or inhibition of the neuronal isoform impaired bacterial clearance, thereby increasing mortality during infection and sepsis [42].

Despite previous applications of L-citrulline in acute toxoplasmosis [28,43], the present study is the first to examine the use of the direct NO precursor, L-arginine, in chronic *T. gondii* infection. As shown in Table 1, L-arginine administration induced a significant reduction (*p* < 0.001 **) in the number of *T. gondii* tissue cysts by a rate of 52.5%. The antiparasitic effects of L-arginine supplementation were also tested in other protozoan infections. In *Trypanosoma cruzi (T. cruzi)* infection, the drug decreased parasitemia levels and heart parasite burden, improving clinical scores and survival in *T. cruzi*-infected mice [44]. Additionally, in *Plasmodium yoelii* infection, pretreatment with L-arginine significantly reduced parasitemia and prolonged survival in infected mice [23]. Conversely, *Plasmodium falciparum* growth was markedly enhanced in L-arginine-supplemented media, whereas L-citrulline supplementation compromised protozoan growth [45]. L-citrulline supplementation in acute toxoplasmosis, especially when combined with sulfamethoxazole-trimethoprim (SMZ-TMP), resulted in a marked reduction of *T. gondii* tachyzoites in mice infected with the virulent RH strain [28]. In a similar context, treating *Giardia intestinalis*-infected rats with L-citrulline resulted in a significant decrease in both trophozoite and cyst stages of the protozoan [46].

The current study identified several parasite-induced changes in brain tissues from *T. gondii*-infected mice (G2), including encephalitis, neuronal loss, and extensive inflammation, findings that align with previous reports for the low-virulent ME-49 *T. gondii* strain [18,20,47]. Building on this, the results showed that L-arginine not only reduced parasitic load but also significantly decreased inflammatory infiltration and improved pathological sequelae during the chronic phase of *T. gondii* infection. In support, L-arginine supplementation has been shown to enhance myocardial inflammatory infiltrates in chronic Chagas disease [48]. The drug also provided protection against *Cryptosporidium parvum* (*C. parvum*) infection. Subcutaneous arginine treatment contributed to improving ileal mucosal histology, promoting growth and weight gain in undernourished suckling mice [49].

Among the intriguing findings in this study are the pathological impacts observed following the selective inhibition of nNOS using 7-NI. Notably, over 50% (57.1%) of the (G4) mice developed severe inflammation (Table 2). Furthermore, comparable pathological effects were reported for 7-NI in animal models challenged with *T. spiralis* [13] and *H. heterophyes* [14]. In these helminth infections, the inhibition of the neuronal enzyme similarly contributed to disrupted tissue architecture, apoptosis, and pronounced inflammatory changes in the intestinal sections of the 7-NI-treated animals. Taken together, these data further confirm the protective role of nNOS during chronic *T. gondii* infection, as its inhibition led to the emergence of parasite-induced sequelae.

In contrast to the positive impacts attributed to the neuronal isotype, several studies have reported detrimental effects for the inducible enzyme iNOS in experimental toxoplasmosis. Nitric oxide generated by iNOS has been shown to induce necrosis in the intestinal mucosa of *T. gondii*- infected mice [15]. Selective iNOS inhibition, on the other hand, led to a significant resolution in inflammatory infiltration within the liver sections of mice treated with aminoguanidine [18]. Additionally, iNOS knockout (iNOS -/-) rats exhibited complete resistance to *T. gondii* infection [38].

Figure 3 shows that *T. gondii* infection induced strong nNOS expression in brain sections of infected mice, indicating upregulation of the marker during chronic toxoplasmosis. Dincel and Atmaca [30] reported similar findings: the number of nNOS-expressing neurons was greater in *T. gondii*-infected mice than in the control group. However, despite the increased neuronal enzyme expression, this was not associated with a corresponding elevation in NO level. Instead, as shown in Figure 4, a significant (*p* < 0.001 **) reduction in NO production was observed following *T. gondii* infection compared to the negative control group (G1). This reduction can be explained by the evasive strategies adopted by *T. gondii* to inhibit nitric oxide production by infected, activated macrophages [17,50].

*Toxoplasma gondii* possesses a putative nitric oxide synthase (NOS) motif within its genomic sequence. As a result, the protozoan is able to produce nitrites using L-arginine as a substrate [51]. Similarly, Furtado et al. [52] reported the first immunolocalization of the constitutive nitric oxide synthase (cNOS)-like enzyme in the promastigote stages of both *Leishmania amazonensis* and *Leishmania braziliensis*. The study suggested that the activity of the cNOS-like enzyme enables the *Leishmania* parasite to produce NO, thereby competing with the host for the arginine substrate.

The local tissue abundance of nNOS observed in brain sections from L-arginine-treated mice (Figure 3d), together with a corresponding elevation in NO levels (Figure 4), provides a mechanistic explanation for the anti-parasitic action of L-arginine reported in this study. Notably, L-arginine-driven nitric oxide production is regarded as a primary killing factor against different protozoan elements, including *T. gondii* [28], *Trypanosoma cruzi* [44], *Plasmodium yoelii* [23], and *Cryptosporidium parvum* [49]. In contrast, selective nNOS blockage in (G4) resulted in the lowest serum NO levels (23.03 Umol/L) (Figure 4). This finding suggests that the neuronal enzyme is a significant source of cerebral NO production during chronic toxoplasmosis.

Although endogenous NO is a major mediator in host defence against infections [8,11], in toxoplasmosis, iNOS-derived NO exhibits opposing roles in mice and humans [8]. While NO demonstrates a protective effect in mice [36], Bando et al. [37] confirmed that it promotes parasitic growth in human hosts.

## 5. Conclusions

In toxoplasmosis, the impacts of nitric oxide have oscillated between friend, foe, and frenemy, reflecting its complex involvement in disease progression. Based on the current study, the originating source and balanced levels of nitric oxide production are critical factors in determining its positive and harmful outcomes. The findings of this report challenge the view that the inducible isotype, iNOS, rather than the neuronal or endothelial enzymes, serves as the primary nitric oxide synthase responsible for effector resistance against the intracellular protozoan *T. gondii*. Instead, the study posits that the neuronal isoenzyme constitutes a significant source of nitric oxide production, contributing to parasitic elimination during the chronic phase of infection. In addition, observations in this work indicate that nNOS-derived nitric oxide exerts protective rather than pathological effects.

### Strengths, Limits, and Future Perspectives

Building on previous insights, this study has several strengths. Notably, it represents the first known use of the direct precursor for NO, L-arginine, in chronic *T. gondii* infection. Second, it provides the initial demonstration of the possible implications of specific nNOS inhibition on cerebral chronic toxoplasmosis. Finally, the study shows that, rather than the overall magnitude of NO, what seems more important is the source of its production, the timing, and the tissue affected. Despite these strengths, certain limitations warrant consideration. The study chiefly focused on the less virulent ME49 *T. gondii* strain. Therefore, it remains to be verified whether the protective impacts of nNOS would also extend to the infection with the more virulent RH strain of the parasite. Additionally, only the brain sections of the tested mice were analyzed, emphasizing the need for further research to determine whether these central nervous system findings are systemic or tissue-specific. Another limitation of the current work is the absence of a DMSO control group in the experimental study design. Further studies are warranted to investigate the potential roles of the different NOS isoforms, specifically iNOS and eNOS, in the brain sections of *T. gondii*-infected mice.

## Figures and Tables

**Figure 1:**
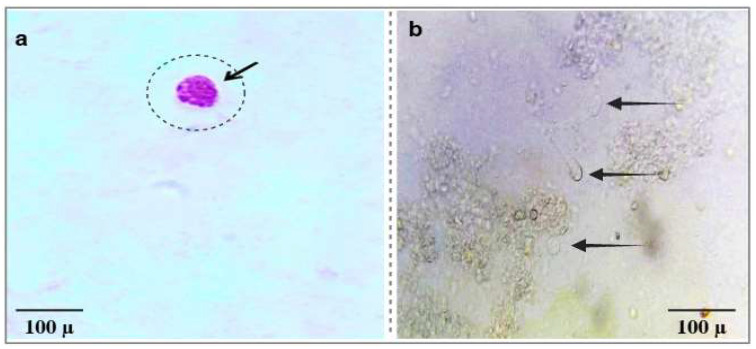
Photomicrographs depicting light microscopic examination of *T. gondii* tissue cysts recovered in brain tissue homogenates from infected mice. (**a**): Infected-untreated mice (G2) showing an encircled *T. gondii* cyst containing numerous bradyzoites (black arrow, Giemsa stain, × 400); (**b**): Infected–7NI-treated mice (G4) with unstained tissue cysts (black arrows, × 200).

**Figure 2:**
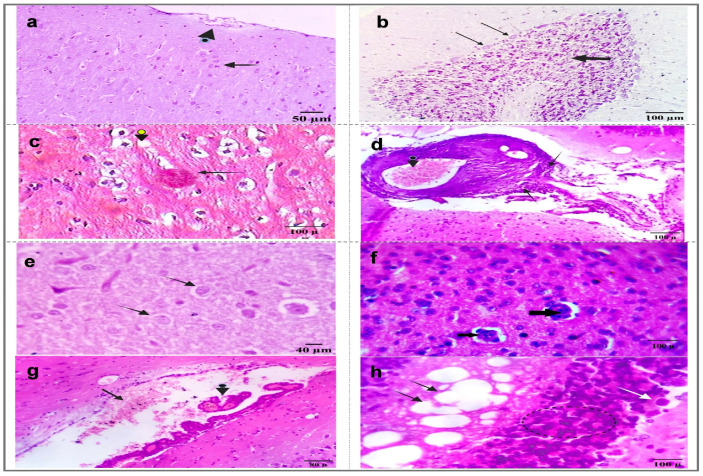
Photomicrographs of brain sections from different study groups. Panels (**a**,**b**) represent G1 (Non-infected, non-treated control group) exhibiting (**a**): normal meningeal structure (arrowhead) and cortical cells (black arrow) (H&E, × 100); (**b**): normal cerebellar Purkinje (fine arrows) and granular cells (broad arrow) (H&E, × 400). Panels (**c**,**d**) represent G2, (*T. gondii*-infected, untreated mice) showing (**c**): *T. gondii* tissue cyst containing many bradyzoites (black arrow) with neuronal loss and vacuolar degeneration (arrowhead) (H&E, × 400); (**d**): encephalitis represented by cerebral vascular congestion with glomeruloid wall appearance (arrowhead) and extensive lymphocytic infiltrations (black arrows) (H&E, × 400); Panels (**e**,**f**) represent G3 (infected–L-arginine-treated mice); (**e**): normal configuration of neuronal cells (black arrows) (H&E, × 200); (**f**): degeneration of *T. gondii* cysts as a result of therapy (black arrows) (H&E, × 400). Panels (**g**,**h**) represent G4 (infected–7NI-treated mice) featuring (**g**): ventricular dilatation due to haemorrhagic fluid accumulation (black arrow), and a hyperemic choroid plexus (arrowhead) (H&E × 200); (**h**): multiple circumscribed malacic areas (black arrows), a degenerated and apoptotic Purkinje cell (white arrow), and disorganized granular cells (black circle) (H&E, × 400).

**Figure 3:**
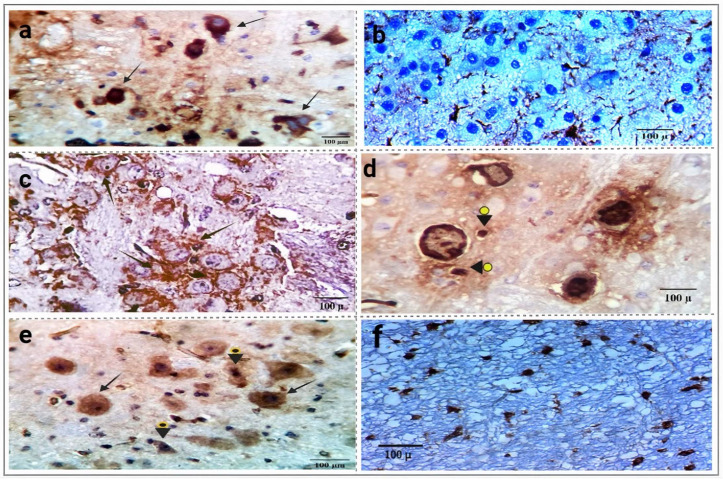
Immunohistochemical expressions of the nNOS isoenzyme in the brain sections from different study groups. Panel (**a**) represents G1 (non-infected, non-treated control group) exhibiting moderate nNOS immunostaining around neuronal cells (black arrows) (IHC, X400). Panel (**b**) shows a control using a secondary antibody without a primary antibody, with no visible staining, further proving experimental specificity (IHC, X200). Panels (**c**,**d**) show G2 (*T. gondii*-infected, untreated mice) featuring strong positive nNOS expression around neuronal (black arrows) and microglial (arrowheads) cells of the brain (**c** and **d**, respectively) (IHC, X400). Panel (**e**) displays G3 (infected–L-arginine-treated mice) with marked nNOS immunoreactivity around astrocytes (arrowheads) and neuronal cells (black arrows) (IHC, ×400). Panel (**f**) presents G4 (infected–7NI-treated mice) exhibiting negative nNOS immunohistochemical staining (IHC, ×200).

**Figure 4:**
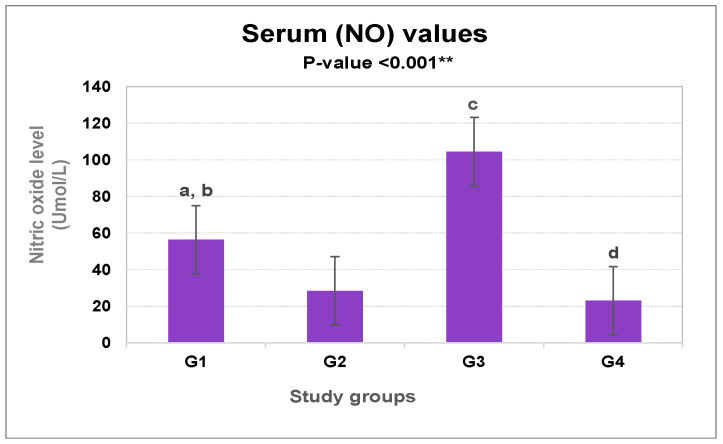
Statistical comparisons of the serum nitric oxide (NO, Umol/L) concentrations among the study groups. Data are presented as means with error bars and were analyzed using ANOVA followed by a post hoc analysis. G1: Non-infected, non-treated group; G2: Infected, untreated group; G3: Infected-L-arginine treated group, and G4: Infected-7-Nitroindazole treated group. Note: “a” indicates a significant difference vs. (G2) (*p* < 0.001 **): *T. gondii* infection induced a significant reduction in NO expression compared to the negative control group (G1), while “b” indicates a significant difference vs. (G4) (*p* < 0.001 **): Selective nNOS blockage in (G4) resulted in the lowest serum NO levels (23.03 Umol/L), showing a significant reduction (*p* < 0.001 **) compared to group (G1), “c” indicates a significant difference vs. (G2) (*p* < 0.001 **), and “d” indicates an insignificant difference vs. (G2) (*p* > 0.05).

**Table 1:** The mean number of *Toxoplasma gondii* tissue cysts in the brain sections of mice from different experimental groups.

Groups	Mean ± SD	Range	Change%	F-Test	*p*-Value	Pairwise Comparisons
G1: Non-infected, non-treated (*n* = 14)	0 ± 0	0	--------	711.5875	<0.001 **	P1: <0.001 **
G2: Infected, untreated group (*n* = 14)	462.4 ± 63.25	336–522	--------			P2: <0.001 **
G3: Infected-L-arginine treated group (*n* = 14)	219.7 ± 32.09	180–287	52.5% reduction			P3: <0.001 **
G4: Infected-7-Nitroindazole treated group (*n* = 14)	675.9 ± 41.65	604–727	46.2% increase			

F: ANOVA test, *p*: Probability, **: Highly significant difference. Post-Hoc analysis was performed after ANOVA test to determine the specific differences between groups; P1: (G2) vs. (G3); P2: (G2) vs. (G4); P3: (G3) vs. (G4).

**Table 2:** Extent of inflammatory infiltration changes in the brain sections of the infected study groups.

Groups	Inflammatory Scoring				χ^2^	*p*-Value	Pairwise Comparisons
	(+1) Mild	(+2) Moderate	(+3) Large	(+4) Severe			
G2: Infected untreated group (n = 14)	0%	21.4%	35.7%	42.9%	35.81	<0.001 **	P1 < 0.001 **
G3: Infected-L-arginine treated group (n = 14)	64.3%	35.7%	0%	0%			P2: 0.3 (NS)
G4: Infected-7-Nitroindazole treated group (n = 14)	0%	0%	42.9%	57.1%			P3: < 0.001 **

(χ2): Chi-square Test; *p*: value for comparing between the studied groups; **: Highly significant difference, NS: Non-significant difference (*p* > 0.05). Post-Hoc analysis was performed to determine the specific differences between groups; P1: (G2) vs. (G3); P2: (G2) vs. (G4); P3: (G3) vs. (G4).

**Table 3:** nNOS Immunohistochemical expression in the brain sections of different study groups.

Groups	nNOS Immunohistochemical Score		
	Negative/Low	Moderate	High
G1: Control negative group (n = 14)	78.6%	21.4%	0.0%
G2: Infected, untreated group (n = 14)	21.4%	35.7%	42.9%
G3: Infected-L-arginine treated group (n = 14)	7.1%	42.9%	50.0%
G4: Infected-7-Nitroindazole treated group (n = 14)	100.0%	0.0%	0.0%
χ^2^ *p *-value Pairwise comparisons	35.257 <0.001 ** P1 < 0.05 * P2: 0.6 (NS) P3: <0.001 ** P4: <0.001 **		

(χ2): Chi-square Test; *p*: value for comparing between the studied groups; * *p* value < 0.05 is significant, ** *p* value < 0.001 is highly significant; NS: Non-significant difference (*p* > 0.05); Post-Hoc analysis was performed to determine the specific differences between groups; P1: (G1) vs. (G2); P2: (G2) vs. (G3); P3: (G1) vs. (G3); P4: (G2) vs. (G4).

## Data Availability

The original contributions presented in this study are included in the article material. Further inquiries can be directed to the corresponding author.

## Abbreviations

The following abbreviations are used in this manuscript:

*T. gondii*	*Toxoplasma gondii*
7-NI	7-Nitroindazole
NO	Nitric oxide
NOS	Nitric oxide synthase
iNOS	Inducible nitric oxide synthase
nNOS	Neuronal nitric oxide synthase
eNOS	Endothelial nitric oxide synthase
cNOS-	Constitutive nitric oxide synthase
DMSO	Dimethyl sulfoxide
IHC	Immunohistochemical
ABC	Avidin-biotin-peroxidase complex
PBS	Phosphate-buffered saline
DAB	Diaminobenzidine
SD	Standard deviation
Th1	T helper-1
IFN-γ	Interferon-gamma
TNF-α	Tumor Necrosis Factor-alpha
*T. spiralis*	*Trichinella spiralis*
*H. heterophyes*	*Heterophyes heterophyes*
*T. cruzi*	*Trypanosoma cruzi*
*P. yoelii*	*Plasmodium yoelii*
*C. parvum*	*Cryptosporidium parvum*

## References

[1] Harker K.S., Ueno N., Lodoen M.B. (2015). *Toxoplasma gondii* dissemination: A parasite’s journey through the infected host. Parasite Immunol..

[2] Farhab M., Aziz M.W., Shaukat A., Cao M.X., Hou Z., Huang S.Y., Li L., Yuan Y.G. (2025). Review of Toxoplasmosis: What We Still Need to Do. Vet. Sci..

[3] Abdelbaset A.E., Abushahba M.F.N., Igarashi M. (2022). *Toxoplasma gondii* in humans and animals in Japan: An epidemiological overview. Parasitol. Int..

[4] Dubey J.P., Frenkel J.K. (1976). Feline toxoplasmosis from acutely infected mice and the development of *Toxoplasma* cysts. J. Protozool..

[5] Kasper L.H., Boothroyd J.C., Warren K., Agabian N. (1992). *Toxoplasma gondii*: Immunology and molecular biology. Immunology of Parasitic Infections.

[6] Bhopale G.M. (2003). Pathogenesis of toxoplasmosis. Comp. Immunol. Microbiol. Infect. Dis..

[7] James S.L. (1995). Role of nitric oxide in parasitic infections. Microbiol. Rev..

[8] Omar M., Abdelal H.O. (2022). Nitric oxide in parasitic infections: A friend or foe?. J. Parasit. Dis..

[9] Wink D.A., Mitchell J.B. (1998). Chemical biology of nitric oxide: Insights into regulatory, cytotoxic, and cytoprotective mechanisms of nitric oxide. Free Radic. Biol. Med..

[10] Ascenzi P., Bocedi A., Gradoni L. (2003). The anti-parasitic effects of nitric oxide. IUBMB Life.

[11] Bredt D.S., Snyder S.H. (1990). Isolation of nitric oxide synthetase, a calmodulin-requiring enzyme. Proc. Natl. Acad. Sci. USA.

[12] Zhou L., Zhu D.Y. (2009). Neuronal nitric oxide synthase: Structure, subcellular localization, regulation, and clinical implications. Nitric Oxide.

[13] Omar M., Fathy G., Mohammed S., El-taib A. (2025). Investigating the dichotomous nature of nitric oxide during the enteral phase of *Trichinella spiralis* infection in mice: An experimental study. Immuno.

[14] Abdel Aziz M., Elsayed H. (2021). Insights into the effects of inducible and neuronal nitric oxide synthase isoenzymes in experimental intestinal heterophyiasis. Parasitol. United J..

[15] Khan I.A., Schwartzman J.D., Matsuura T., Kasper L.H. (1997). A dichotomous role for nitric oxide during Acute *Toxoplasma gondii* infection in mice. Proc. Natl. Acad. Sci. USA.

[16] Scharton-Kersten T.M., Yap G., Magram J., Sher A. (1997). Inducible nitric oxide is essential for host control of persistent but not acute infection with the intracellular pathogen *Toxoplasma gondii*. J. Exp. Med..

[17] Cabral G.R.A., Wang Z.T., Sibley L.D., Da Matta R.A. (2018). Inhibition of nitric oxide production in activated macrophages caused by *Toxoplasma gondii* infection occurs by distinct mechanisms in different mouse macrophage cell lines. Front. Microbiol..

[18] Omar M., Abaza B.E., Mousa E., Ibrahim S.M., Rashed H.E., Farag T.I. (2021). Effect of spiramycin versus aminoguanidine and their combined use in experimental toxoplasmosis. J. Parasit. Dis..

[19] Djurković-Djaković O., Milenković V., Nikolić A., Bobić B., Grujić J. (2002). Efficacy of atovaquone combined with clindamycin against murine infection with a cystogenic (Me49) strain of *Toxoplasma gondii*. J. Antimicrob. Chemother..

[20] Eraky M.A., El-Fakahany A.F., El-Sayed N.M., Abou-Ouf E.A., Yaseen D.I. (2016). Effects of Thymus vulgaris ethanolic extract on chronic toxoplasmosis in a mouse model. Parasitol. Res..

[21] Giarcia L.S., Bruckner D.A., Giboda M.N., Vokurkova P., Kopacek O. (1977). Macroscopic and microscopic examination of fecal specimens. Diagnostic Medical Parasitology.

[22] Viana M.L., Dos Santos Rd Generoso Sde V., Nicoli J.R., Martins Fdos S., Nogueira-Machado J.A., Arantes R.M., Correia M.I., Cardoso V.N. (2013). The role of L-arginine-nitric oxide pathway in bacterial translocation. Amino Acids.

[23] Zheng L., Pan Y., Feng Y., Cui L., Cao Y. (2015). L-Arginine supplementation in mice enhances NO production in spleen cells and inhibits *Plasmodium yoelii* transmission in mosquitoes. Parasit. Vectors.

[24] Perotti C.A., Nogueira M.S., Antunes-Rodrigues J., Cárnio E.C. (1999). Effects of a neuronal nitric oxide synthase inhibitor on lipopolysaccharide-induced fever. Braz. J. Med. Biol. Res..

[25] Salerno L., Sorrenti V., Di Giacomo C., Romeo G., Siracusa M.A. (2002). Progress in the development of selective nitric oxide synthase (NOS) inhibitors. Curr. Pharm. Des..

[26] Dubey J.P., Ferreira L.R., Alsaad M., Verma S.K., Alves D.A., Holland G.N., McConkey G.A. (2016). Experimental toxoplasmosis in rats induced orally with eleven strains of *Toxoplasma gondii* of seven genotypes: Tissue tropism, tissue cyst size, neural lesions, tissue cyst rupture without reactivation, and ocular lesions. PLoS ONE.

[27] Drury R.A.B., Wallington E.A. (1980). Carleton’s Histological Technique.

[28] Saad A.E., Zoghroban H.S., Ghanem H.B., El-Guindy D.M., Younis S.S. (2023). The effects of L-citrulline adjunctive treatment of *Toxoplasma gondii* RH strain infection in a mouse model. Acta Trop..

[29] Czarnewski P., Araújo E.C.B., Oliveira M.C., Mineo T.W.P., Silva N.M. (2017). Recombinant TgHSP70 immunization protects against *Toxoplasma gondii* brain cyst formation by enhancing inducible nitric oxide expression. Front. Cell. Infect. Microbiol..

[30] Dincel G.C., Atmaca H.T. (2015). Nitric oxide production increases during *Toxoplasma gondii* encephalitis in mice. Exp. Parasitol..

[31] Hao X.P., Pretlow T.G., Rao J.S., Pretlow T.P. (2001). Inducible nitric oxide synthase (iNOS) is expressed similarly in multiple aberrant crypt foci and colorectal tumors from the same patients. Cancer Res..

[32] Chan Y.H. (2003). Biostatistics 102: Quantitative data—Parametric & non-parametric tests. Singap. Med. J..

[33] Hosking B.C., Watson T.G., Leathwick D.M. (1996). Multigeneric resistance to oxfendazole by nematodes in cattle. Vet. Rec..

[34] Leslie E., Geoffrey J., James M., Kirkpatrick L.A., Feeney B.C. (1991). Statistical analysis. Interpretation and Uses of Medical Statistics.

[35] Schlüter D., Deckert-Schlüter M., Lorenz E., Meyer T., Röllinghoff M., Bogdan C. (1999). Inhibition of inducible nitric oxide synthase exacerbates chronic cerebral toxoplasmosis in *Toxoplasma gondii*-susceptible C57BL/6 mice but does not reactivate the latent disease in *T. gondii*-resistant BALB/c mice. J. Immunol..

[36] Alexander J., Scharton-Kersten T.M., Yap G., Roberts C.W., Liew F.Y., Sher A. (1997). Mechanisms of Innate Resistance to *Toxoplasma gondii* Infection. Philos. Trans. R. Soc. Lond. B Biol. Sci..

[37] Bando H., Lee Y., Sakaguchi N., Pradipta A., Ma J.S., Tanaka S., Cai Y., Liu J., Shen J., Nishikawa Y. (2018). Inducible nitric oxide synthase is a key host factor for *Toxoplasma* GRA15-dependent disruption of the gamma interferon-induced antiparasitic human response. mBio.

[38] Wang Z.J., Yu S.M., Gao J.M., Zhang P., Hide G., Yamamoto M., Lai D.H., Lun Z.R. (2021). High resistance to *Toxoplasma gondii* infection in inducible nitric oxide synthase knockout rats. iScience.

[39] Li E., Zhou P., Singer S.M. (2006). Neuronal nitric oxide synthase is necessary for elimination of *Giardia lamblia* infections in mice. J. Immunol..

[40] Dincel G.C., Kul O. (2015). Increased expressions of ADAMTS-13, neuronal nitric oxide synthase, and neurofilament correlate with severity of neuropathology in Border disease virus-infected small ruminants. PLoS ONE.

[41] Fatemi S.H., Sidwell R., Akhter P., Sedgewick J., Thuras P., Bailey K., Kist D. (1998). Human influenza viral infection in utero increases nNOS expression in hippocampi of neonatal mice. Synapse.

[42] Cui X., Besch V., Khaibullina A., Hergen A., Quezado M., Eichacker P., Quezado Z.M. (2007). Neuronal nitric oxide synthase deficiency decreases survival in bacterial peritonitis and sepsis. Intensive Care Med..

[43] Zheng C., Lin J. (1998). The role of L-arginine and L-citrulline in activated macrophage against *Toxoplasma gondii* infection in vitro. Zhongguo Ji Sheng Chong Xue Yu Ji Sheng Chong Bing Za Zhi.

[44] Carbajosa S., Rodríguez-Angulo H.O., Gea S., Chillón-Marinas C., Poveda C., Maza M.C., Colombet D., Fresno M., Gironès N. (2018). L-arginine supplementation reduces mortality and improves disease outcome in mice infected with *Trypanosoma cruzi*. PLoS Negl. Trop. Dis..

[45] Awasthi V., Chauhan R., Chattopadhyay D., Das J. (2017). Effect of L-arginine on the growth of *Plasmodium falciparum* and immune modulation of host cells. J. Vector Borne Dis..

[46] Zoghroban H.S., Ibrahim F.M., Nasef N.A., Saad A.E. (2023). The impact of L-citrulline on murine intestinal cell integrity, immune response, and arginine metabolism in the face of *Giardia lamblia* infection. Acta Trop..

[47] Atmaca H.T., Kul O., Karakuş E., Terzi O.S., Canpolat S., Anteplioğlu T. (2014). Astrocytes, microglia/macrophages, and neurons expressing Toll-like receptor 11 contribute to innate immunity against encephalitic *Toxoplasma gondii* infection. Neuroscience.

[48] Bianchini Narde M., Belli Cassa Domingues E.L., Ribeiro Gonçalves K., Lomar Viana M., Santos Zanini M., Geraldo de Lima W., Bahia M.T., Matos Dos Santos F. (2021). L-arginine supplementation increases cardiac collagenogenesis in mice chronically infected with Berenice-78 *Trypanosoma cruzi* strain. Parasitol. Int..

[49] Castro I.C., Oliveira B.B., Slowikowski J.J., Coutinho B.P., Siqueira F.J., Costa L.B., Sevilleja J.E., Almeida C.A., Lima A.A., Warren C.A. (2012). Arginine decreases *Cryptosporidium parvum* infection in undernourished suckling mice involving nitric oxide synthase and arginase. Nutrition.

[50] Butcher B.A., Fox B.A., Rommereim L.M., Kim S.G., Maurer K.J., Yarovinsky F., Herbert D.R., Bzik D.J., Denkers E.Y. (2011). *Toxoplasma gondii* rhoptry kinase ROP16 activates STAT3 and STAT6 resulting in cytokine inhibition and arginase-1-dependent growth control. PLoS Pathog..

[51] Gutierrez-Escobar A.J., Arenas A.F., Villoria-Guerrero Y., Padilla-Londoño J.M., Gómez-Marin J.E. (2008). *Toxoplasma gondii*: Molecular cloning and characterization of a nitric oxide synthase-like protein. Exp. Parasitol..

[52] Furtado R.R., Soares D.C., Prado A.F., Farias L.H.S., Da Silva B.J.M., Rodrigues A.P.D., Silva E.O. (2021). Constitutive nitric oxide synthase-like enzyme in two species involved in cutaneous and mucocutaneous leishmaniasis. Parasitol. Int..

